# High-power, very-high-power, and low-power radiofrequency ablation for atrial fibrillation: A Bayesian network meta-analysis

**DOI:** 10.1016/j.hroo.2026.02.018

**Published:** 2026-02-28

**Authors:** Eduardo Celentano, Ernesto Cristiano, Barbara Ignatiuk, Martina Renda, Anthea D’Amico, Elena Bia, Raffaele Rainone, Eduardo Urgesi, Giuseppe Grandinetti, Roberto Lorusso, Natasja M.S. De Groot

**Affiliations:** 1 Electrophysiology Unit, Humanitas Gavazzeni Hospital, Bergamo, Italy; 2 Translational Electrophysiology Unit, Department of Cardiology, Erasmus MC, University Medical Center Rotterdam, The Netherlands; 3 Cardiovascular Research Institute Maastricht, Maastricht University, Maastricht, The Netherlands; 4 Electrophysiology Unit, Mater Dei Hospital, Bari, Italy

**Keywords:** Atrial fibrillation, Catheter ablation, Radiofrequency ablation, High- and very-high-power short-duration, Network meta-analysis

## Abstract

**Background:**

High-power short-duration (HPSD) and very-high-power short-duration (vHPSD) radiofrequency (RF) strategies have been proposed to improve lesion quality and procedural efficiency in atrial fibrillation ablation.

**Objective:**

This study aimed to compare HPSD, vHPSD, and low-power long-duration (LPLD) ablation by performing a systematic review and Bayesian network meta-analysis.

**Methods:**

Following Cochrane/Preferred Reporting Items for Systematic Reviews and Meta-Analyses 2020 guidance, multiple databases were searched, and 51 studies (13,751 patients) were included. Consistent and unrelated-mean-effects models were fitted, inconsistency and design-by-treatment tests assessed coherence, and leave-one-out and Egger’s analyses explored robustness and small-study effects. The primary outcome was atrial arrhythmia recurrence, and secondary outcomes included procedural safety (in-hospital complications) and efficiency parameters (procedure, fluoroscopy, and RF time). Treatment ranking was derived using the surface under the cumulative ranking curve.

**Results:**

Both HPSD and vHPSD reduced recurrence compared with LPLD (rate ratios [RR] 0.85; 95% credible interval [CrI] 0.75–0.96; and RR 0.79; 95% CrI 0.64–0.96, respectively), whereas the vHPSD vs HPSD comparison was inconclusive (RR 0.93; 95% CrI 0.75–1.13). Ranking suggested vHPSD as having the highest probability of being the better choice for procedural outcomes (79% vHPSD, 21% HPSD, 0% LPLD). Safety was comparable among RF strategies. Procedure and RF times were shorter with high-power approaches (approximately 34 minutes shorter); HPSD had the shortest fluoroscopy time (−7 minutes vs LPLD), and vHPSD had the shortest RF time (−24 minutes vs LPLD).

**Conclusion:**

High-power RF strategies were associated with improved procedural efficiency and, in the overall evidence base, lower recurrence than conventional LPLD; however, recurrence benefit was mainly supported by observational evidence. Comparative safety showed no significant differences. Adequately powered randomized trials are warranted to clarify long-term outcomes and directly compare HPSD and vHPSD.

## Graphical abstract

**Figure:**
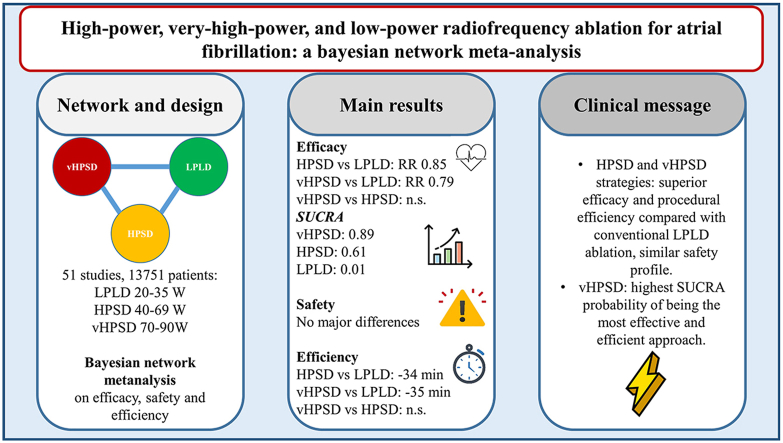

**Key Findings**

▪ In a Bayesian network meta-analysis of 51 studies (13,751 patients), both high-power short-duration (HPSD) and very-high-power short-duration (vHPSD) radiofrequency (RF) ablation strategies were associated with lower atrial arrhythmia recurrence than conventional low-power long-duration (LPLD) ablation.
▪ Compared with LPLD, recurrence was reduced with HPSD (rate ratio [RR] 0.85) and vHPSD (RR 0.79); the vHPSD vs HPSD comparison was inconclusive.
▪ Composite in-hospital complications were comparable across strategies.
▪ High-power approaches shortened procedure time by ∼35 minutes vs LPLD (HPSD mean difference [MD] −34.4 minutes; vHPSD MD −34.7 minutes); vHPSD achieved the shortest RF delivery time (MD −24.1 minutes vs LPLD), whereas HPSD achieved the shortest fluoroscopy time (MD −6.9 minutes vs LPLD).
▪ Ranking analyses suggested that vHPSD had the highest probability of being the best for efficacy (surface under the cumulative ranking curve [SUCRA] 0.89; 79%) and RF delivery time (SUCRA 1.00; 100%), whereas HPSD ranked the best for fluoroscopy time (SUCRA 0.94; 88%).

## Introduction

Catheter ablation is central to rhythm control in symptomatic, drug-refractory atrial fibrillation (AF), most often accomplished through pulmonary vein isolation (PVI).^1^ Low-power long-duration (LPLD) (20–35 W, 20–30 s/lesion) radiofrequency (RF) delivery has evolved toward high-power short-duration (HPSD) (45–70 W, 5–10 s/lesion) and very-high-power short-duration (vHPSD) modalities (70–90 W, 4–7 s/lesion).^2^ These procedures emphasize resistive over conductive heating; produce rapid, contiguous lesions with less collateral injury; and potentially reduce procedure and fluoroscopy times while preserving safety.^3^, ^4^, ^5^, ^6^

Comparative evidence remains fragmented across designs and RF catheter technologies, especially comparing HPSD and vHPSD. Therefore, we undertook a comprehensive Bayesian network meta-analysis (NMA) comparing HPSD, vHPSD, and LPLD to clarify their relative efficacy, safety, and efficiency.

## Methods

### Protocol and registration

The review followed Preferred Reporting Items for Systematic Reviews and Meta-Analyses (PRISMA) 2020 guidelines^7^ and the Cochrane Handbook for Systematic Reviews.^8^ The systematic review and meta-analysis protocol was registered in the International Prospective Register of Systematic Reviews under protocol CRD420251005450. Additional methods information, recurrence, and complication definition are presented in the Supplemental Material.

### Ethics statement

This study is a systematic review and meta-analysis based exclusively on published literature. Only aggregated data extracted from publicly available publications were used; no new data were collected, and no individual participant data were accessed. Therefore, ethical approval and informed consent were not required.

### Search strategy and study selection

The literature search strategy was executed from 2000 to June 2025 across various databases, including PubMed, the Cochrane Library, Embase, Web of Science, and Scopus, ensuring a thorough exploration of the scientific literature. Only English-language articles were used for this review. An extensive array of keywords was carefully selected and applied to guarantee inclusivity, encompassing facets such as “radiofrequency ablation,” “high-power,” “very high-power,” “short-duration,” “low-power,” “long-duration,” “atrial fibrillation,” and “catheter ablation.” A summary of the detailed search strategy, including specific combinations of keywords and operators, is presented in Supplemental Table 1. To uphold the integrity of the process and minimize potential selection bias, 2 independent researchers (E.U. and Er.Cr.) explored the literature, resolving disagreements through consensus. A third researcher (Ed.Ce.) was engaged in cases of persistent disagreement to ensure resolution and reliability.

Only randomized and observational comparative studies reporting direct comparisons between at least 2 of the predefined power–duration strategies (LPLD, HPSD, and/or vHPSD) were eligible for inclusion. Single-arm studies were excluded from the study.

**Figure 1:**
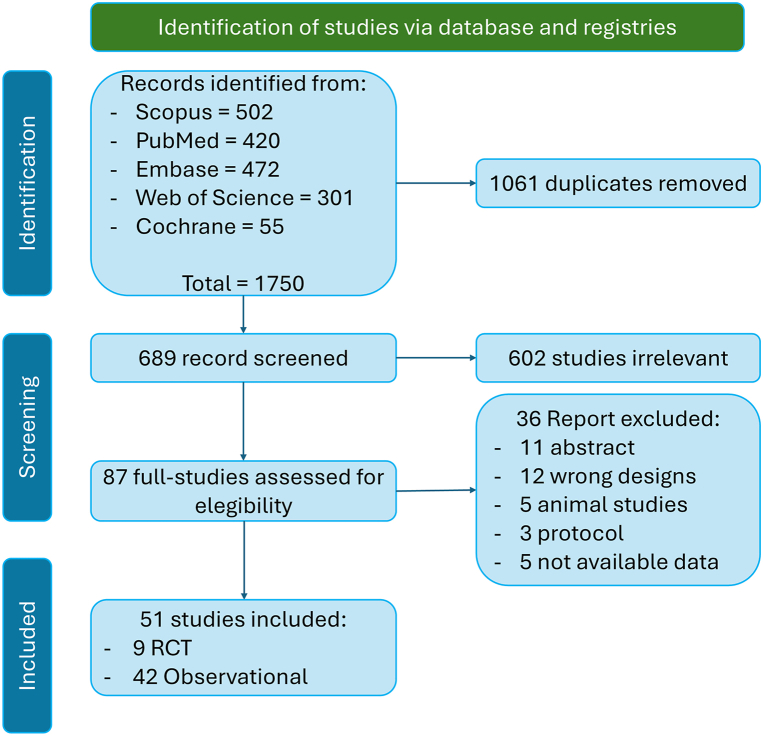
Preferred Reporting Items for Systematic Reviews and Meta-Analyses flow diagram of study selection for the Bayesian network meta-analysis comparing low-power long-duration, high-power short-duration, and very-high-power short-duration radiofrequency ablation strategies in patients with atrial fibrillation. The diagram shows the number of records identified through database searching and other sources; the studies screened, assessed for eligibility, and excluded with reasons; and the final 51 studies (13,751 patients) included in the quantitative synthesis. RCT = randomized controlled trial.

The PRISMA flow diagram summarizing the study selection process is presented in Figure 1, and the PRISMA checklist is presented in Supplemental Table 2.

### Eligibility criteria

The population, intervention, comparison, and outcome framework was adapted to design specific eligibility criteria (Supplemental Table 3).^9^ Randomized and observational studies enrolling adults (≥18 years) undergoing AF ablation with HPSD, vHPSD, or LPLD strategies were eligible if they reported at least 1 prespecified clinical or electrophysiological outcome. Studies using non-RF energy sources, extreme power settings (>90 W), or animal data or lacking adequate outcome reporting were excluded.

### Data extraction and quality assessment

2 reviewers (E.U. and Er.Cr.) independently extracted study, patient, and outcome data. Randomized trials were evaluated using the Cochrane Risk of Bias 2 tool, and cohort studies using the Risk Of Bias in Non-randomized Studies of Interventions (ROBINS-I).

Data included sample size, follow-up duration, baseline patient characteristics, publication year, RF power usage, baseline antiarrhythmic drug use, left ventricular ejection fraction, body mass index, left atrial diameter, and AF type and adjunctive procedural protocol features such as intraprocedural PVI evaluation, waiting time, PVI protocol, and technology information (for more details, see Supplemental Materials). Where studies reported the median and interquartile range, we estimated the standard deviation using the interquartile range/1.35 approximation recommended by the Cochrane Handbook.^8^ As a sensitivity analysis, we applied the sample size adjusted estimator of Wan et al.^10^^,^^11^

### Outcomes

The primary efficacy outcome was atrial arrhythmia recurrence (AF, atrial tachycardia, and atrial flutter) at the longest available follow-up. Because follow-up duration varied across studies, recurrence was analyzed primarily using incidence rate ratios (RRs) (events per person-time), a standard approach for comparing incidence rates under unequal observation times. As a sensitivity analysis addressing concerns about fixed-time outcome definitions, we also analyzed recurrence as a binary endpoint and estimated odds ratios (ORs). Secondary outcomes included early complications (pericardial effusion, tamponade, stroke, transient ischemic attack [TIA], phrenic palsy, or atrioesophageal fistula) and procedural efficiency (procedure, fluoroscopy, and RF times). Exploratory safety outcome was analyzed as early periprocedural complications, defined as events occurring during hospitalization or within the periprocedural window as reported by each study (most commonly in-hospital). Because delayed adverse events were inconsistently captured and variably defined, long-term safety outcomes were not pooled.

### Statistical analysis

**Figure 2:**
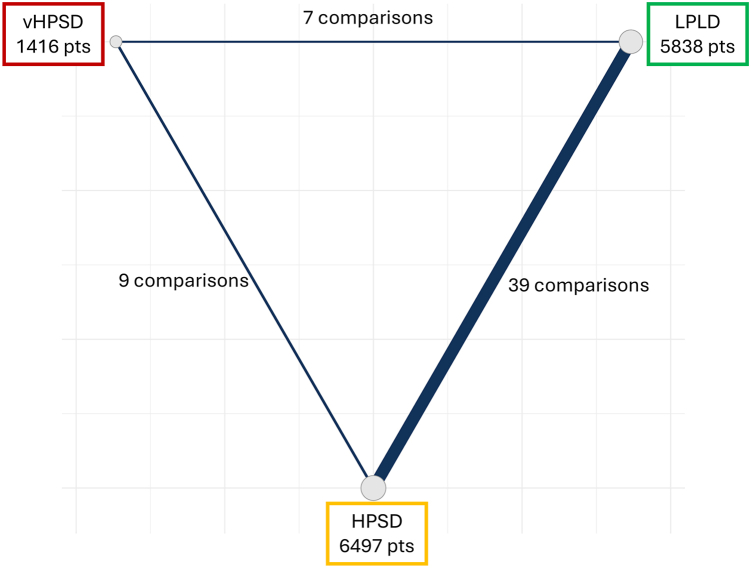
Network of treatment comparisons in the Bayesian network meta-analysis. Nodes represent the ablation strategies evaluated—conventional LPLD, HPSD, and vHPSD radiofrequency ablation in patients with atrial fibrillation—with node size proportional to the total number of patients assigned to each strategy. *Lines* between nodes indicate direct head-to-head comparisons, with *line thickness* reflecting the number of studies contributing to each comparison. HPSD = high-power short-duration; LPLD = low-power long-duration; vHPSD = very-high-power short-duration.

A Bayesian random-effects NMA was conducted comparing vHPSD, HPSD, and LPLD strategies. The network of treatment comparisons is presented in Figure 2. For the Bayesian model, weak informative priors were applied to all model parameters to facilitate estimation while constraining inferences to plausible values based on general background knowledge. The primary outcome was modeled as a Poisson likelihood with a log-link. Relative effects were expressed as RRs with 95% credible intervals (CrIs). Treatment ranking was derived from posterior distributions using the surface under the cumulative ranking curve (SUCRA).

Between-study heterogeneity was modeled as a normal random effect with between-trial SD τ. Inconsistency was evaluated using node-splitting and design-by-treatment tests; model fit was compared using the deviance information criterion.

For binary safety outcomes, binomial likelihoods with logit links were used, and results expressed as ORs. Continuous efficiency outcomes were analyzed as mean differences under a normal–identity model using arm means and standard errors.

### Meta-regression

Prespecified meta-regressions evaluated effect modification to exclude possible covariate effects on main outcomes. The tested covariate were age (per 10 years), body mass index (per 5 kg/m^2^), left atrial diameter (per 5 mm), left ventricular ejection fraction (per 5%), male sex, hypertension, diabetes, heart failure, previous stroke/TIA (per 10% of patients), and CHA_2_DS_2_-VASc score (per 1 point).

Continuous covariates were standardized for interpretability. Predictions of treatment effects across covariate values were obtained from fitted Bayesian network meta-regressions.

### Sensitivity analyses

Sensitivity analyses included (1) Bayesian network meta-regressions for year of publication, (2) separate networks for randomized and observational studies, (3) alternative priors and covariates scaling, (4) subanalysis for PVI-only ablation protocol trials, and (5) fixed-time outcome considering recurrence as a binary endpoint and estimated ORs. Small-study effects were assessed using Egger’s regression test. To explore the potential impact of small-study effects, we performed a sensitivity analysis restricted to larger studies (>100 patients). Model adequacy was verified through trace plots and effective sample sizes. Leave-one-out refits and posterior predictive checks ensured the robustness of the model.

### Software

Analyses were performed in R version 4.5.1 using the multinma package for NMA and network meta-regression. For data processing and visualization, the dplyr, tidyr, and ggplot2 R packages were also used.

## Results

### Study selection and characteristics

51 studies comprising 13,751 patients were included. Of these, 9 were randomized controlled trials (RCTs)^12^, ^13^, ^14^, ^15^, ^16^, ^17^, ^18^, ^19^, ^20^ and 42 were observational cohort studies^5^^,^^21^, ^22^, ^23^, ^24^, ^25^, ^26^, ^27^, ^28^, ^29^, ^30^, ^31^, ^32^, ^33^, ^34^, ^35^, ^36^, ^37^, ^38^, ^39^, ^40^, ^41^, ^42^, ^43^, ^44^, ^45^, ^46^, ^47^, ^48^, ^49^, ^50^, ^51^, ^52^, ^53^, ^54^, ^55^, ^56^, ^57^, ^58^, ^59^, ^60^, ^61^ (Figures 1 and 2). The mean age ranged from 57 to 68 years, 65% were male, and the average left atrial diameter was 42 ± 5 mm.

Ablation power settings clustered as follows (for more details about power/duration/catheter technology, see Supplemental Table 4):

• LPLD: 20–35 W, approximately 20–30 seconds per lesion
• HPSD: 40–69 W, approximately 5–10 seconds per lesion
• vHPSD: 70–90 W, approximately 4–7 seconds per lesion

Procedural techniques varied across studies, but endpoint definitions and follow-up duration (median 12 months) were consistent (Table 1).

**Table 1:** Baseline clinical, echocardiographic, and procedural characteristics of the studies included in the network meta-analysis, stratified by ablation strategy (LPLD, HPSD, and vHPSD RF ablation)

Authors	Year of publication	RCT	Comparison	RF power (W)	Sample size	AF type (PAF)	Mean age	Sex (male)	Mean CHADSVASC	LVEF (%)	Mean LAD (mm)	Follow-up time (mo)	Blanking period (d)	Arrhythmia recurrence	Complication	Procedure time (min)
Mansour et al^21^	2004	No	LPLD vs HPSD	25/50	40/40	33/32	53/55	33/35	-/-	-/-	39/41	21/11	0	16/10	3/4	-/-
Nilsson et al^22^	2006	No	LPLD vs HPSD	30/45	45/45	32/26	51/55	36/36	1/2	-/-	-/-	15/15	30	12/11	1/1	-/-
Yamada et al^23^	2006	No	LPLD vs HPSD	30/40	47/61	47/61	56/59	40/51	-/-	67/65	35/35	6/6	90	22/20	0/0	223/152
Kanj et al^12^	2007	Yes	LPLD vs HPSD	35/50	60/61	-/-	62/59	49/51	-/-	54/54	44/40	6/6	60	19/11	0/13	-/-
Matiello et al^24^	2008	Yes	LPLD vs HPSD	30/40	42/179	27/110	54/52	29/143	-/-	-/-	41/42	14/15	90	27/82	3/13	-/-
Winkle et al^5^	2011	No	HPSD vs vHPSD	40/70	742/101	234/36	62/59	539/72	-/-	-/-	43/43	12/12	90	416/61	10/4	129/217
Baher et al^25^	2018	No	LPLD vs HPSD	25/50	113/574	80/276	68/69	67/385	2/3	-/-	-/-	30/30	90	46/241	0/0	251/149
Okamatsu et al^13^	2019	No	LPLD vs HPSD	20/50	40/20	31/13	66/65	26/13	2/2	64/65	40/40	10/6	90	1/1	1/0	163/-
Pambrun et al^26^	2019	No	LPLD vs HPSD	25/40	50/50	50/50	62/65	30/35	-/-	-/-	-/-	12/12	0	6/5	0/0	107/73
Bunch et al^27^	2020	No	LPLD vs HPSD	30/50	402/402	190/202	66/67	253/262	-/-	55/55	-/-	36/36	90	67/90	0/0	171/104
Ejima et al^28^	2020	No	LPLD vs HPSD	30/50	60/60	60/60	66/63	42/44	2/2	57/58	-/-	21/21	60	17/7	0/0	140/119
Kottmaier et al^29^	2020	No	LPLD vs vHPSD	30/70	100/97	100/97	61/61	60/57	2/2	55/57	-/-	12/12	45	35/37	17/13	111/90
Kumagai and Toyama^30^	2020	No	LPLD vs HPSD	20/50	80/80	24/30	63/63	66/60	1/1	62/62	-/-	19/19	90	19/11	0/0	85/65
Kyriakopoulou et al^31^	2020	No	LPLD vs HPSD	35/40	105/80	105/80	64/67	65/47	2/2	-/-	44/43	12/12	90	14/8	0/0	111/91
Leo et al^14^	2020	Yes	LPLD vs HPSD	20/40	40/40	15/16	57/61	33/26	-/-	60/59	48/42	29/29	90	19/9	0/2	180/165
Shin et al^15^	2020	Yes	LPLD vs HPSD	30/40	50/100	24/48	59/58	33/81	2/2	59/57	41/41	12/12	90	11/17	0/1	162/122
Yavin et al^32^	2020	No	LPLD vs HPSD	20/45	112/112	67/76	65/62	79/71	3/2	58/60	47/44	23/14	30	34/23	2/0	-/-
Yazaki et al^33^	2020	No	LPLD vs HPSD	20/50	32/32	29/22	66/61	20/27	-/-	56/55	-/-	10/10	60	11/14	0/0	150/115
Chen et al^34^	2021	No	LPLD vs HPSD	25/40	40/40	25/30	57/57	29/26	2/2	66/64	35/36	11/11	90	13/7	0/0	124/91
Dikdan et al^35^	2021	No	LPLD vs HPSD	20/50	51/76	21/32	61/63	40/54	1/2	56/57	-/-	12/12	0	13/21	1/0	101/71
Francke et al^16^	2021	No	LPLD vs HPSD	20/50	20/100	9/49	66/66	7/60	3/3	60/54	41/41	3/3	0	5/6	0/3	109/80
Hansom et al^36^	2021	No	LPLD vs HPSD	20/50	107/107	60/67	62/62	81/69	2/2	-/-	41/41	12/12	90	29/23	3/1	309/229
O'Brien et al^37^	2021	No	LPLD vs HPSD	35/50	93/88	60/39	64/64	64/57	1/2	-/-	39/40	12/12	90	16/17	0/1	140/121
Okamatsu et al^38^	2021	No	LPLD vs HPSD	20/40	301/1032	172/583	67/68	210/716	2/2	62/62	41/41	12/12	90	57/193	4/25	180/153
Park et al^39^	2021	No	LPLD vs HPSD	20/40	945/315	553/180	59/59	699/232	2/2	62/63	42/43	12/12	90	825/280	36/10	181/135
Wielandts et al^17^	2021	Yes	LPLD vs HPSD	35/45	48/48	48/48	61/64	33/32	1/1	-/-	40/39	6/6	60	4/5	0/0	103/85
Cheng et al^40^	2022	No	LPLD vs HPSD	30/45	36/36	36/36	63/59	20/27	2/2	62/63	38/38	3/3	0	5/9	4/4	145/112
Cui et al^41^	2022	No	LPLD vs HPSD	30/50	43/49	28/29	58/62	34/39	1/1	62/60	38/38	21/21	90	9/6	0/0	-/-
Ding et al^42^	2022	No	LPLD vs HPSD	35/50	20/20	10/2	65/66	13/13	2/2	-/-	40/45	12/12	90	1/6	0/0	143/120
Hijioka et al^43^	2022	No	LPLD vs HPSD	20/45	73/85	53/46	62/64	55/64	-/-	61/61	43/42	24/24	90	19/22	2/0	193/172
Liu et al^44^	2022	No	LPLD vs HPSD	35/45	60/74	51/57	66/67	37/46	3/3	57/62	38/41	12/12	90	17/5	3/1	34/25
Mueller et al^45^	2022	No	HPSD vs vHPSD	50/90	42/42	19/18	67/68	15/24	-/-	54/55	-/-	12/12	90	14/16	74/7	85/89
Salló et al^46^	2022	No	LPLD vs HPSD vs vHPSD	30/50/90	53/50/53	29/37/31	60/58/68	32/27/33	2/2/3	60/55/56	44/43/43	9/9/9	0	19/5/4	2/0/0	85/79/70
Seidl et al^47^	2022	No	HPSD vs vHPSD	50/90	21/31	15/26	64/58	17/25	1/2	59/59	40/38	23/23	90	7/13	1/2	121/106
Vassallo et al^48^	2022	No	LPLD vs HPSD	20/45	158/182	121/106	53/58	113/131	2/2	63/61	41/43	27/33	90	30/30	0/0	145/92
Heeger et al^62^	2023	No	HPSD vs vHPSD	40/90	50/50	24/26	66/67	31/34	-/-	-/-	-/-	12/12	90	21/16	3/1	101/59
Jin et al^50^	2023	No	LPLD vs HPSD	25/40	308/308	308/308	60/60	187/194	2/2	65/65	36/36	12/12	90	48/61	5/5	-/-
Lee et al^18^	2023	Yes	LPLD vs HPSD	25/50	31/29	17/17	63/67	25/20	2/2	60/60	-/-	12/12	90	11/3	0/0	286/236
Manukyan et al^51^	2023	No	LPLD vs HPSD	20/50	98/98	46/57	60/64	66/58	2/3	-/-	-/-	12/12	90	26/11	2/2	98/73
O'Neill et al^19^	2023	Yes	HPSD vs vHPSD	35/90	90/90	75/64	62/64	59/61	2/2	-/-	40/39	6/6	90	15/15	1/0	75/70
Popa et al^52^	2023	No	LPLD vs vHPSD	30/60	541/574	253/254	65/65	352/355	2/2	56/55	-/-	12/12	45	298/255	58/38	155/122
Sousa et al^63^	2023	No	LPLD vs HPSD	25/40	80/80	80/80	62/61	53/44	1/2	60/60	40/41	36/36	90	15/14	0/0	100/80
Zhu and Lin^53^	2023	No	LPLD vs HPSD	30/40	100/123	72/86	62/60	53/64	3/3	58/58	42/43	12/12	90	25/28	0/2	-/-
Bortone et al^54^	2024	No	HPSD vs vHPSD	50/50	75/75	75/75	68/69	51/45	2/2	60/60	-/-	12/12	90	7/5	0/0	65/61
Compagnucci et al^55^	2024	No	LPLD vs vHPSD	35/50	40/40	0/0	62/63	33/35	-/-	51/55	-/-	23/23	90	17/10	0/0	198/81
Fink et al^56^	2024	No	LPLD vs HPSD vs vHPSD	30/50/90	102/102/103	37/35/34	68/65/67	67/70/71	2/3/2	51/51/52	46/46/46	17/17/17	90	30/26/26	2/1/2	105/81/70
Joza et al^57^	2024	No	LPLD vs HPSD	25/40	225/173	225/173	60/62	163/104	1/2	-/-	42/42	12/12	90	50/52	8/4	254/152
Keegan et al^58^	2024	No	LPLD vs vHPSD	30/70	128/82	82/36	60/64	90/59	1/2	-/-	-/-	13/13	90	49/14	10/7	180/125
Mitrzak et al^59^	2024	No	LPLD vs vHPSD	35/90	54/54	36/40	57/58	37/36	-/-	-/-	-/-	3/3	0	20/14	0/1	145/120
Szegedi et al^20^	2024	Yes	HPSD vs vHPSD	50/90	22/24	15/11	63/64	13/11	-/-	51/52	-/-	12/12	90	1/5	0/0	84/76
Yazaki et al^60^	2025	No	LPLD vs HPSD	30/50	665/304	475/182	62/62	510/235	1/1	55/53	-/-	24/24	60	259/95	12/5	169/123

Studies are listed in chronological order and the table reports study design (RCT or observational), RF power settings, total sample size per treatment arm, AF type with the number of patients with PAF, mean age, proportion of male patients, mean CHA_2_DS_2_-VASc score, LVEF, LAD, follow-up duration, and crude numbers of atrial arrhythmia recurrences, complications, and procedure times for each treatment group.

AF = atrial fibrillation; HPSD = high-power short-duration; LAD = left atrial diameter; LPLD = low-power long-duration; LVEF = left ventricular ejection fraction; PAF = paroxysmal atrial fibrillation; RCT = randomized controlled trial; RF = radiofrequency; vHPSD = very-high-power short-duration.

Procedural protocol features are presented in Supplemental Table 4. Across the 51 included studies, 30 (58.8%) adopted a PVI-only lesion set, whereas 21 (41.2%) allowed additional ablation beyond PVI. When reported, intraprocedural waiting time was typically 20–30 minutes. PVI durability was most commonly assessed by bidirectional block (35 of 51; 68.6%), whereas provocative testing to detect dormant conduction was performed in 25 of 51 studies (49.0%) (adenosine in 20 and isoproterenol in 13). Most studies included patients with paroxysmal or persistent AF who underwent PVI using predominantly irrigated-tip catheters (49 of 51; 96.1%). Contact-force (CF) sensing was reported in 42 of 51 studies (82.4%) overall and became prevalent in contemporary cohorts (41 of 44 [93.2%] from 2019 onward vs 0 of 7 before 2019). Ablation-index guidance was reported in 38 of 51 studies (74.5%) overall and almost exclusively in studies published from 2019 onward (36 of 44 [81.8%] vs 0 of 7 before 2019). Notably, comparisons involving vHPSD were almost entirely contemporary (13 of 14 published between 2020 and 2024).

### Study quality

Risk of bias was assessed separately for RCTs using the Cochrane Risk of Bias 2 tool and for nonrandomized studies of interventions using ROBINS-I. Across the 9 randomized trials, most domains were judged to be at low risk of bias. Between 55.6% and 66.7% of studies were at low risk for the randomization process, deviations from intended interventions, missing outcome data, and outcome measurement, whereas 88.9% of trials raised some concerns regarding the selection of the reported result. Overall, 8 RCTs (88.9%) were rated as having “some concerns” and 1 trial (11.1%) as at high risk of bias based on at least 1 high-risk domain (Supplemental Figure 1). In contrast, the 42 nonrandomized studies were generally at higher risk of bias: when assessed using ROBINS-I, 25 studies (59.5%) were judged at high overall risk and the remaining 17 (40.5%) as having some concerns. High risk of bias among nonrandomized studies was driven predominantly by confounding, for which 59.5% of studies were rated high risk, whereas missing data and outcome measurement were most often judged to raise some concerns (66.7% and 81.0% of studies, respectively). Classification of interventions was consistently judged to be at low risk of bias, but nearly all studies (97.6%) raised some concerns about the selection of the reported results (Supplemental Figure 2).

### Primary efficacy outcome

**Figure 3:**
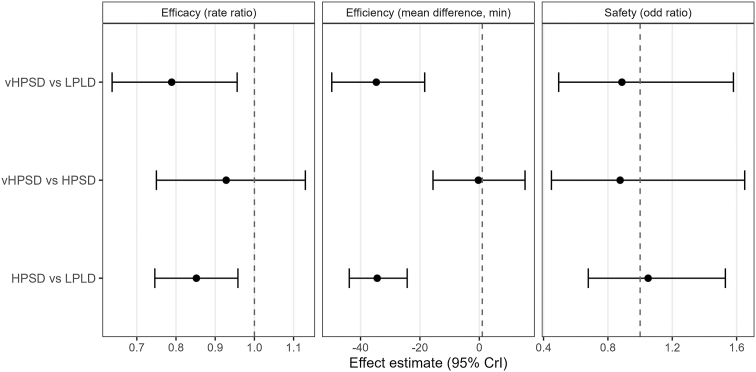
Forest plots of the Bayesian network meta-analysis for primary efficacy, safety, and procedural efficiency outcomes. For each comparison among conventional LPLD, HPSD, and vHPSD radiofrequency (RF) ablation strategies in patients with atrial fibrillation (AF), *squares* represent posterior median rate ratios (RRs) or odds ratios (ORs) for atrial arrhythmia recurrence and the composite of in-hospital complications, and posterior median mean differences (MDs) (minutes) for procedure time, RF delivery time, and fluoroscopy time, with *horizontal lines* indicating the corresponding 95% CrIs. Values of RRs and ORs <1 and negative MDs indicate a benefit of HPSD or vHPSD compared with LPLD. CrI = credible interval; HPSD = high-power short-duration; LPLD = low-power long-duration; vHPSD = very-high-power short-duration.

**Figure 4:**
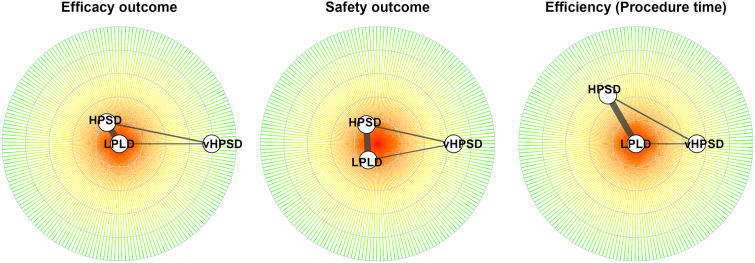
Surface under the cumulative ranking curve (SUCRA) values and rank probabilities for each ablation strategy in the Bayesian network meta-analysis. In these radial SUCRA plots, *bars* depict SUCRA values (range 0–100) and the estimated probability of being the best treatment for primary efficacy (freedom from atrial arrhythmia recurrence), safety (composite of in-hospital complications), and procedural efficiency outcomes (procedure time, radiofrequency [RF] delivery time and fluoroscopy time) for conventional LPLD, HPSD, and vHPSD RF ablation in patients with atrial fibrillation. Higher SUCRA values indicate a greater likelihood that a given strategy ranks as the most favorable treatment for the corresponding outcome. HPSD = high-power short-duration; LPLD = low-power long-duration; vHPSD = very-high-power short-duration.

In the Bayesian NMA, both HPSD (RR 0.85) and vHPSD (RR 0.79) were associated with a significantly reduced atrial arrhythmia recurrence compared with LPLD, with no significant difference between the 2 high-power strategies (Table 2 and Figure 3). SUCRA rankings for efficacy were 0.89 for vHPSD (79% probability of being best), 0.61 for HPSD (21%), and 0.01 for LPLD (0%) (Table 3 and Figure 4).

**Table 2:** Pairwise Bayesian network meta-analysis estimates for primary efficacy, safety, and procedural efficiency outcomes according to ablation strategy

Efficacy outcome		
Treatment comparisons	Rate ratio	95% CrI (2.5%–97.5%)
HPSD vs LPLD	0.85	0.75–0.96
vHPSD vs LPLD	0.79	0.64–0.96
vHPSD vs HPSD	0.93	0.75–1.13

Safety outcome		
Treatment comparisons	Rate ratio	95% CrI (2.5%–97.5%)
Treatment comparisons	Odd ratio	95% CrI (2.5%–97.5%)
HPSD vs LPLD	1.05	0.68–1.53
vHPSD vs LPLD	0.89	0.49–1.58
vHPSD vs HPSD	0.88	0.45–1.65

Efficiency outcome		
Treatment comparisons	Mean difference (min)	95% CrI (2.5%–97.5%)
Procedure time		
HPSD vs LPLD	−34.4	−43.8 to −24.3
vHPSD vs LPLD	−34.7	−49.7 to −18.4
vHPSD vs HPSD	−0.3	−15.6 to 15.4
RF delivery time		
HPSD vs LPLD	−16.0	−20.3 to −11.6
vHPSD vs LPLD	−24.1	−31.0 to −17.6
vHPSD vs HPSD	−8.2	−14.3 to −2.0
Fluoroscopy time		
HPSD vs LPLD	−6.9	−11.6 to −2.3
vHPSD vs LPLD	−2.7	−9.7 to 4.2
vHPSD vs HPSD	4.2	−2.6 to 11.0

Rate ratios with 95% CrIs are reported for the primary efficacy outcome of atrial arrhythmia recurrence, odds ratios for the composite of in-hospital complications, and mean differences (minutes) for procedure time, RF delivery time, and fluoroscopy time. Values of rate ratios and odds ratios <1 and negative mean differences indicate a benefit of HPSD or vHPSD radiofrequency ablation compared with conventional LPLD ablation.

CrI = credible interval; HPSD = high-power short-duration; LPLD = low-power long-duration; RF = radiofrequency; vHPSD = very-high-power short-duration.

**Table 3:** Ranking of ablation strategies based on SUCRA values and probability of being the best treatment for each outcome in the Bayesian network meta-analysis

Treatment	SUCRA	Probability of being the best
Efficacy outcome		
vHPSD	0.89	79%
HPSD	0.61	21%
LPLD	0.01	0%
Safety outcome		
vHPSD	0.73	65%
HPSD	0.36	19%
LPLD	0.41	16%
Procedure time		
vHPSD	0.76	52%
HPSD	0.74	48%
LPLD	0.00	0%
RF delivery time		
vHPSD	1.00	100%
HPSD	0.50	0%
LPLD	0.00	0%
Fluoroscopy time		
vHPSD	0.46	11%
HPSD	0.94	88%
LPLD	0.10	0%

SUCRA values (range 0–1) and corresponding probabilities (%) are reported for efficacy (freedom from atrial arrhythmia recurrence), safety (composite of in-hospital complications), and procedural efficiency outcomes (procedure time, RF delivery time, and fluoroscopy time) for conventional LPLD, HPSD, and vHPSD RF ablation. Higher SUCRA values indicate a greater likelihood that a given strategy is the most favorable treatment for the corresponding outcome.

HPSD = high-power short-duration; LPLD = low-power long-duration; RF = radiofrequency; SUCRA = surface under the cumulative ranking curve; vHPSD = very-high-power short-duration.

Between-study heterogeneity was low to moderate (τ = 0.21 [95% CrI 0.05–0.36]; τ^2^ = 0.04 [95% CrI 0.003–0.13]).

### Safety outcomes

No significant differences in safety outcomes were observed among ablation strategies (Table 2). According to SUCRA safety rankings, vHPSD had 65% probability of being the best choice, followed by HPSD with 19% probability and LPLD with 16% (Table 3 and Figure 4). Between-study heterogeneity was moderate (τ = 0.44; 95% CrI 0.04–0.93).

Typical adverse events were pericardial effusion (0.5%–0.8%) and vascular complications (0.3%–0.6%), followed by stroke/TIA (0.2%) and atrioesophageal fistula (<0.1%).

### Procedural efficiency

Compared with LPLD, both high-power ablation approaches substantially shortened procedure and RF delivery times (Table 2). HPSD achieved the shortest fluoroscopy time, with an 88% probability of being the best procedure for this metric, whereas vHPSD had the shortest RF delivery time and 100% probability of being the most effective ablation modality (Table 3). Shortening of procedure time was similar between HPSD (48% probability of being best) and vHPSD (52%) (Table 3 and Figure 4).

### Sensitivity analyses

**Figure 5:**
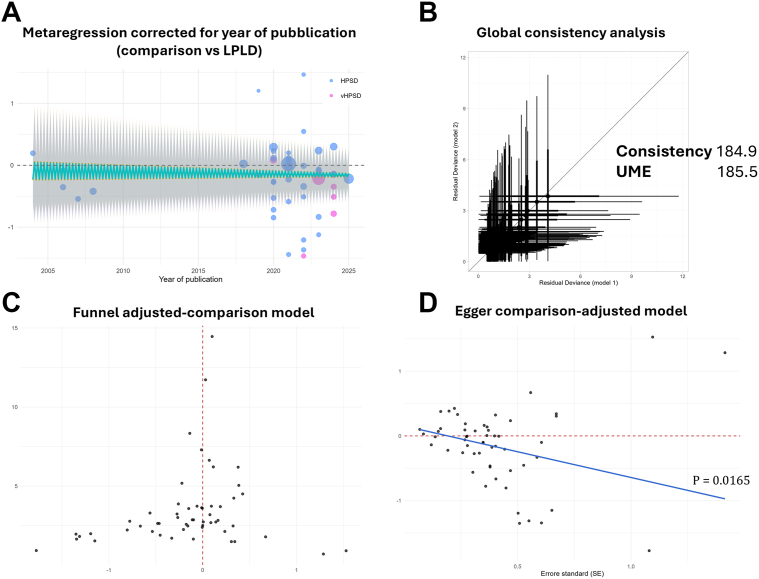
Meta-regression, global consistency, and small-study effect analyses for the Bayesian network meta-analysis of the primary efficacy outcome in patients with atrial fibrillation. **A:** Random-effects meta-regression of the log rate ratio (RR) for atrial arrhythmia recurrence comparing HPSD or vHPSD radiofrequency (RF) ablation with conventional LPLD RF ablation according to calendar year of publication; *circles* indicate study-specific effects, and the *solid line with shaded area* denotes the fitted regression and its 95% credible interval. **B:** Global consistency assessment plotting residual deviance from the consistency model (x-axis) against the UMEs model (y-axis), with similar deviance values supporting overall agreement between direct and indirect evidence. **C:** Comparison-adjusted funnel plot of study-specific treatment effects (log RR) for the primary efficacy outcome, centered on the overall network estimate (*vertical dashed line*), used to explore small-study effects and publication bias. **D:** Comparison-adjusted Egger’s regression of treatment effect on its SE, with the *solid line* indicating the fitted regression and the reported *P* value testing for funnel-plot asymmetry. HPSD = high-power short-duration; LPLD = low-power long-duration; SE = standard error; vHPSD = very-high-power short-duration; UME = unrelated-mean-effects.

Meta-regressions evaluating effect modification by prespecified parameters did not identify a significant impact of any parameter (Table 4 and Figure 5A).

**Table 4:** Study-level Bayesian network meta-regression for the primary efficacy outcome of atrial arrhythmia recurrence

Covariate	β [vHPSD vs HPSD] (95% CrI)	β [HPSD vs LPLD] (95% CrI)	β [vHPSD vs LPLD] (95% CrI)
Age per 10 y	−0.36 (−0.88 to 0.18)	−0.18 (−0.45 to 0.08)	−0.36 (−0.88 to 0.18)
BMI per 5 kg/m^2^	0.40 (−0.62 to 1.46)	−0.22 (−0.55 to 0.09)	0.40 (−0.62 to 1.46)
LAD per 5 mm	−0.13 (−0.84 to 0.54)	−0.04 (−0.15 to 0.08)	−0.13 (−0.84 to 0.54)
LVEF per 5 %	−0.12 (−0.78 to 0.55)	0.01 (−0.19 to 0.20)	−0.12 (−0.78 to 0.55)
Study year per 10 y	−0.21 (−0.70 to 0.26)	−0.04 (−0.26 to 0.19)	−0.21 (−0.70 to 0.26)
% male per 10%	−0.15 (−0.42 to 0.12)	−0.01 (−0.14 to 0.12)	−0.15 (−0.42 to 0.12)
Hypertension per 10%	0.06 (−0.20 to 0.29)	0.00 (−0.11 to 0.09)	0.06 (−0.20 to 0.29)
Diabetes per 10%	0.25 (−0.34 to 0.91)	−0.03 (−0.26 to 0.19)	0.25 (−0.34 to 0.91)
Heart failure per 10%	0.05 (−0.60 to 0.69)	0.03 (−0.15 to 0.22)	0.05 (−0.60 to 0.69)
Previous stroke per 10%	0.91 (−1.32 to 3.27)	0.51 (−0.39 to 1.44)	0.91 (−1.32 to 3.27)
CHA_2_DS_2_-VASc score per 1 point	−0.52 (−1.37 to 0.25)	−0.05 (−0.34 to 0.21)	−0.52 (−1.37 to 0.25)

Regression coefficients (β) with 95% CrIs are reported for the comparisons between vHPSD and HPSD, between HPSD and LPLD, and between vHPSD and LPLD radiofrequency ablation, according to prespecified covariates (age per 10 years, BMI per 5 kg/m^2^, LAD per 5 mm, LVEF per 5%, study year per 10 years, and proportions per 10% of male sex, hypertension, diabetes, heart failure, previous stroke, and CHA_2_DS_2_-VASc score per 1 point). β coefficients express the change in the estimated treatment effect per specified increment in each covariate, with CrIs that include 0 indicating no statistically credible effect modification.

BMI = body mass index; CrI = credible interval; LAD = left atrial diameter; HPSD = high-power short-duration; LPLD = low-power long-duration; LVEF = left ventricular ejection fraction; vHPSD = very-high-power short-duration.

Global and local inconsistency diagnostics confirmed model coherence (deviance information criterion 184.9 vs 185.5; all node-split *P* > .25) (see Figure 5B and Supplemental Table 5 for global and Supplemental Table 6 for local inconsistency tests). Evidence of small-study effects was observed (Egger’s *P* = .0165). However, in sensitivity analysis restricted to larger studies (>100 patients), the recurrence reduction vs LPLD remained statistically credible (HPSD vs LPLD RR 0.846, 95% CrI 0.722–0.964; vHPSD vs LPLD RR 0.764, 95% CrI 0.584–0.959) (Figure 5C and 5D, Supplemental Tables 5 and 7).

Leave-one-out sensitivity analysis confirmed base estimates stable across refits (HPSD vs LPLD 0.835 - 0.872; vHPSD vs LPLD 0.759 - 0.831) (see Supplemental Table 8).

Prespecified design-stratified analyses showed that the estimated reduction in recurrence vs LPLD was driven by observational studies (HPSD vs LPLD RR 0.787, 95% CrI 0.629–0.951; vHPSD vs LPLD RR 0.889, 95% CrI 0.779–0.990). In contrast, RCT-only estimates were not conclusive (HPSD vs LPLD RR 0.587, 95% CrI 0.296–1.030; vHPSD vs LPLD RR 1.130, 95% CrI 0.260–3.610). Finally, in the subanalysis restricted to PVI-only trials and fixed-time recurrence, both high-power strategies remained consistent with lower arrhythmia recurrence vs LPLD (Table 5).

**Table 5:** Design-stratified and sensitivity subgroup analyses for the primary efficacy outcome (atrial arrhythmia recurrence)

Contrast	RR	95% CrI 2.5%	95% CrI 97.5%
RCT			
HPSD vs LPLD	0.587	0.296	1.030
vHPSD vs LPLD	1.130	0.260	3.610
Observational			
HPSD vs LPLD	0.787	0.629	0.951
vHPSD vs LPLD	0.889	0.779	0.990
PVI-only strategy			
HPSD vs LPLD	0.730	0.531	0.950
vHPSD vs LPLD	0.700	0.449	0.951
Larger trial only (>100 patients)			
HPSD vs LPLD	0.846	0.722	0.964
vHPSD vs LPLD	0.764	0.584	0.959
Fixed-time recurrence definition (reported as odds ratios)			
HPSD vs LPLD	0.743	0.617	0.872
vHPSD vs LPLD	0.675	0.488	0.894

Results are shown as RRs with 95% CrIs for HPSD vs LPLD and vHPSD vs LPLD, reported separately for RCTs and observational nonrandomized interventional studies and for prespecified sensitivity subsets (PVI-only strategy, larger trials [>100 patients], and fixed-time recurrence definitions). RR <1 indicates a lower recurrence rate with HPSD or vHPSD than LPLD.

CrI = credible interval; HPSD = high-power short-duration; LPLD = low-power long-duration; PVI = pulmonary vein isolation; RCT = randomized controlled trial; RR = rate ratio; vHPSD = very-high-power short-duration.

## Discussion

In this Bayesian NMA including 51 studies and 13,751 patients, most studies were nonrandomized (42 of 51). Both HPSD and vHPSD radiofrequency ablation modalities were associated with a lower atrial arrhythmia recurrence rate than LPLD ablation (HPSD vs LPLD, RR 0.85, 95% CrI 0.75–0.96; vHPSD vs LPLD, RR 0.79, 95% CrI 0.64–0.96), with no statistically significant difference between vHPSD and HPSD (RR 0.93; 95% CrI 0.75–1.13). vHPSD ranked highest for efficacy (SUCRA 0.89 with a 79% probability of being best), followed by HPSD (SUCRA 0.61) and LPLD (SUCRA 0.01). However, the certainty of the recurrence benefit is limited by study design: in prespecified design-stratified analyses, the signal was primarily supported by observational studies, whereas RCT-only estimates were inconclusive and did not demonstrate a clear benefit. Furthermore, although ranking metrics and SUCRA suggested a hierarchy, between-strategy differences were not statistically credible and event rates were low; therefore, these findings should be interpreted as hypothesis generating with respect to long-term comparative effectiveness and safety. Taken together with concordant gains in procedural efficiency and comparable safety, these findings suggest an ongoing paradigm shift in RF energy delivery for AF ablation toward resistive-dominant heating using high-power strategies.

### Historical background

Over the past 3 decades, catheter-based ablation for AF has undergone a profound technological evolution. Early procedures relied on point-by-point LPLD RF delivery using nonirrigated or first-generation irrigated catheters, achieving PVI at the cost of lengthy procedures, substantial fluoroscopy exposure, and variable lesion durability.^61^ The subsequent introduction of CF sensing, ablation indices, and high-resolution electroanatomic mapping improved lesion contiguity and operator confidence, but remained constrained by predominantly conductive heating and relatively long energy application times.^64^ In parallel, single-shot cryoballoon technologies^65^ and minimally invasive surgical or hybrid approaches^66^^,^^67^ were developed to standardize lesion sets and reduce operator dependence, albeit at the price of reduced flexibility and higher procedural complexity. More recently, HPSD RF strategies and subsequently vHPSD protocols enabled by temperature-controlled catheters have deliberately shifted the balance toward resistive-dominant heating, aiming to create wider, shallower, and more homogeneous atrial tissue lesions in a shorter time.^68^ In this landscape, pulsed-field ablation (PFA) has emerged as a nonthermal modality with a distinct safety profile, but long-term lesion durability and comparative effectiveness vs optimized high-power RF remain incompletely defined.^69^^,^^70^ Over the last 2 decades, the technology underpinning RF catheter ablation has changed substantially. The earliest LPLD series were conducted in the mid-2000s, largely before the widespread adoption of CF-sensing catheters, lesion indices, and systematic esophageal temperature monitoring. In contrast, most HPSD and virtually all vHPSD studies have been performed in a more contemporary setting, combining advanced catheter designs, real-time feedback on contact and temperature, and refined electroanatomic mapping workflows. These technological and procedural advances, together with operator experience, are likely to act as important effect modifiers and may amplify the apparent differences between LPLD and high-power strategies when evidence from different eras is synthesized in a single NMA.

### Efficacy and lesion formation

Both HPSD and vHPSD achieved superior long-term arrhythmia-free survival compared with LPLD. The improvement likely reflects the formation of more homogeneous, contiguous atrial lesions and reduced conductive heat loss. Leshem et al^3^ demonstrated that rapid resistive heating produces shallower but wider lesions, promoting durable PVI while minimizing collateral damage. Our pooled SUCRA ranking suggests vHPSD as the most probable effective approach (SUCRA 0.89 with a 79% probability of being the best), followed by HPSD and LPLD.

Although effect sizes vs LPLD were modest, they were consistent across the network and robust to sensitivity analyses, whereas the comparison between vHPSD and HPSD remained statistically inconclusive. This result suggests a plateau of efficacy once the energy per site exceeds approximately 60–70 W for 5–7 seconds.^52^ This threshold aligns with biophysical models indicating the optimal balance between resistive heating and tissue conductivity.^4^^,^^64^^,^^71^

### Safety profile

Despite higher instantaneous power, both HPSD and vHPSD exhibited comparable complication rates with LPLD. Across strategies, safety outcomes were comparable (HPSD vs LPLD OR 1.05, 95% CrI 0.68–1.53; vHPSD vs LPLD OR 0.89, 95% CrI 0.49–1.58; vHPSD vs HPSD OR 0.88, 95% CrI 0.45–1.65), with wide CrIs reflecting the low incidence of severe complications. Incidence of pericardial effusion, stroke/TIA, and atrioesophageal injury was low across studies.^20^^,^^46^^,^^56^^,^^59^^,^^60^ Temperature-controlled and CF-guided ablation systems likely mitigate overheating risk.^61^^,^^72^^,^^62^ Importantly, no esophageal perforations were reported in prospective randomized trials applying vHPSD with real-time luminal temperature monitoring.^12^, ^13^, ^14^, ^15^, ^16^, ^17^, ^18^, ^19^, ^20^ This reinforces the principle that power alone does not characterize safety: duration, contact, and cooling are equally critical. However, given the very low absolute rates of atrioesophageal fistula and stroke/TIA, the network could be underpowered to detect small but clinically relevant differences in rare complications. Furthermore, our safety end point reflects mainly in-hospital/periprocedural reporting and does not capture delayed complications; thus, safety comparisons are exploratory and require confirmation in large prospective registries with standardized follow-up.

### Procedural efficiency

Both high-power approaches meaningfully shortened procedure and RF delivery times. By limiting conductive heat spread and reducing convective cooling, HPSD and vHPSD achieve effective atrial lesion transmurality within seconds.^3^^,^^5^^,^^29^ These efficiency gains are consistent with biophysical models showing that resistive-dominant, short-duration applications achieve transmural lesions within seconds, thereby reducing cumulative catheter dwell time and the likelihood of microdislodgements along the lesion set.^73^^,^^74^ Clinically, the reduction of 30–35 minutes in overall procedure time and 20–25 minutes in RF time is not negligible, especially in centers with high ablation volume or hybrid workflows.^75^ These gains align with data from the Q-FFICIENCY trial,^68^ confirming the reproducibility of this procedural benefit.

### Comparison with previous NMAs

2 NMAs have previously addressed the comparative efficacy and safety of different power–duration strategies for AF ablation.^76^^,^^77^ Our work is concordant with these studies in the overall rationale, evaluating whether high-power strategies improve rhythm outcomes and procedural efficiency compared with low-power approaches, yet differs in several clinically and methodologically relevant aspects that may explain differences in effect estimates. First, node definitions were more stringent in our analysis. Tokavanich et al^76^ defined vHPSD as >50 W, potentially grouping heterogeneous protocols (including intermediate settings) within the vHPSD node, whereas we prespecified vHPSD as 70–90 W and HPSD as 45–70 W to better isolate truly very-high-power protocols aligned with contemporary temperature-controlled workflows. Similarly, Junarta et al^77^ adopted a 70–90 W definition for vHPSD but used broader low-power/long-duration ranges, which may increase within-node heterogeneity. Second, our study provides an update to the evidence base; previous searches extended to September 2022 (Tokavanich et al^76^) and December 2022 (Junarta et al^77^), whereas our review incorporates 13 additional comparative studies published thereafter, reflecting rapid technological evolution (including newer catheter generations and temperature-controlled systems) and changes in procedural workflows. Third, outcome synthesis differed. Previous NMAs primarily analyzed freedom from atrial arrhythmia using ORs at specific follow-up time points; we modeled atrial arrhythmia recurrence using RRs, a strategy that may better accommodate heterogeneous follow-up durations and monitoring intensity across studies. Finally, we expanded the analytical scope by jointly evaluating efficacy, in-hospital safety end points, and procedural efficiency (procedure, RF, and fluoroscopy time) and implementing comprehensive coherence and robustness diagnostics (global/local inconsistency assessments, design-by-treatment interaction, and sensitivity analyses). Collectively, these differences position our NMA as both a methodologically distinct synthesis and a contemporary update, which likely accounts for quantitative discrepancies while maintaining interpretive continuity with previous studies.

### Comparison with PFA

PFA has recently emerged as a nonthermal modality offering rapid lesion creation and high safety margins. Although PFA was not included in the present network, recent meta-analyses suggest that vHPSD achieves similar acute efficacy but remains dependent on tissue resistivity and contact parameters.^69^ Although PFA may reduce collateral damage, its lesion durability beyond 12 months remains less established.^70^ Thus, vHPSD represents a pragmatic, mature alternative in centers without PFA capability, maintaining compatibility with existing RF systems.

### Clinical implications

These findings suggest that high-power strategies may be considered to improve procedural efficiency in experienced centers with experience in HPSD/vHPSD workflows and access to CF- and temperature-controlled catheters. Both HPSD and vHPSD provide improved atrial lesion quality, shorter procedures, and similar safety. vHPSD may offer incremental efficiency gains and potentially enhance lesion durability, particularly when coupled with temperature control and contiguous lesion mapping.

In conclusion, our findings suggest the following practical approach:

• vHPSD may be considered when procedural efficiency is prioritized and contemporary temperature-controlled workflows are available.
• HPSD may represent a pragmatic alternative where vHPSD systems are unavailable.
• LPLD may remain an option in specific scenarios, recognizing that comparisons across eras may be confounded by technology and workflow.

Future studies should focus on integrating artificial intelligence-guided ablation indices with temperature-controlled feedback, exploring ultrashort-pulse paradigms (<4 seconds), and directly comparing vHPSD and PFA for long-term durability and safety outcomes.

### Limitations

Several limitations should be acknowledged. First, network estimates integrate heterogeneous study designs, patient populations, ablation workflows, and follow-up durations, and most of the evidence derives from nonrandomized studies, which are inherently prone to selection bias and confounding. The significant Egger’s test suggested small-study effects, which may inflate modest effect sizes. However, the estimates were consistent in sensitivity analyses restricted to larger studies (>100 patients), supporting the robustness of the study size. Despite this, certainty was downgraded for suspected reporting bias/small-study effects and findings should be interpreted cautiously. Second, the definitions of atrial arrhythmia “recurrence” and “complications,” as well as monitoring strategies, follow-up intensity, and rare complications (such as atrioesophageal fistula), were not fully standardized across trials. This is a well-recognized limitation of the AF ablation literature that potentially leads to misclassification and under- or overestimation of event rates that cannot be fully adjusted using study-level data. Third, although we used subgroup and network meta-regression analyses to adjust for key study-level covariates, residual confounding from unmeasured or inconsistently reported factors (such as operator and center experience, mapping systems, catheter technology generations, and lesion indices) may persist. In particular, we explored the year of publication in meta-regression; year is only a surrogate for multiple correlated innovations and therefore cannot fully remove technology-related confounding. Fourth, the assumptions of transitivity and consistency underlying NMA could be challenged by the uneven distribution of contemporary technologies (eg, temperature-controlled catheters and advanced CF guidance) across HPSD, vHPSD, and LPLD arms. Indeed, although global and local inconsistency checks did not reveal major incoherence, small violations cannot be excluded. Fifth, safety outcomes were relatively infrequent and often incompletely reported, resulting in wide CrIs and limited statistical power to detect differences in rare but critical complications such as atrioesophageal fistula or stroke/TIA. Therefore, our safety findings should be interpreted as exploratory findings. Finally, the number and size of RCTs directly comparing vHPSD with HPSD remain limited, long-term follow-up beyond 12–24 months is sparse, and most studies were conducted in high-volume electrophysiology centers. Because catheter technology and lesion-guidance strategies were tightly linked to the study era and incompletely reported, we could not include them as covariates in the meta-regression; therefore, confounding by technology cannot be fully excluded when comparing LPLD with high-power strategies. These aspects may restrict the generalizability of these results and underscore the need for large-scale randomized trials and prospective real-world registries.

## Conclusion

HPSD and vHPSD ablation strategies yield superior efficacy and procedural efficiency compared with LPLD ablation, while maintaining a similar safety profile. In the overall evidence base, recurrence was lower than that of LPLD; however, this result was largely supported by observational studies and should be interpreted cautiously. Ranking suggests vHPSD as having the highest probability of being the better choice for procedural outcomes. These findings endorse high-power radiofrequency ablation as the preferred thermal energy delivery strategy for AF ablation, pending confirmatory randomized evidence comparing vHPSD with HPSD and PFA. Further adequately powered randomized trials are needed to define comparative long-term effectiveness and safety.

## Disclosures

The authors have no conflicts of interest to disclose.

## Footnotes

Appendix Supplementary data associated with this article can be found in the online version at https://doi.org/10.1016/j.hroo.2026.02.018.
