# Supplementary material for: High-power, very-high-power, and low-power radiofrequency ablation for atrial fibrillation: A Bayesian network meta-analysis
Source: Heart Rhythm O2. 2026 Feb 28;7(5):848–60. doi: 10.1016/j.hroo.2026.02.018 (PMC13198348; doi:10.1016/j.hroo.2026.02.018)

**Supplementary Materials of “High-power, very-high-power, and low-power radiofrequency ablation for atrial fibrillation: a bayesian network meta-analysis”**

***Index***

**Methods page 2**

Eligibility Criteria page 2

Quality Assessment page 2

Atrial arrhythmia recurrence page 3

Procedural complications page 3

Statistical considerations page 3

Table S1. Search Strategies page 4

Table S2. PRISMA NMA Checklist page 5

Table S3 PICO page 12

**Baseline information page 13**

Table S4 Procedural characteristics page 13

**Sensitivity analyses section page 16**

Table S5 Global fit page 16

Table S6 Local inconsistency page 16

Table S7 Egger test page 16

Table S8 Leave-one-out page 16

**Supplementary Figures page 17**

Figure S1. Risk of bias (RoB2) page 17

Figure S2. Risk of bias (ROBINS-I) page 18

**METHODS**

**Eligibility Criteria**

For the inclusion criteria, studies had to meet the following criteria: (1) The study population consisted of atrial fibrillation patients; (2) the intervention group received one type of ablation intervention (low power long duration (LPLD) radiofrequency, high power short duration (HPSD) radiofrequency, very high power short duration (vHPSD) radiofrequency; (3) Used LPLD radiofrequency or another type of ablation as a control group; (4) the outcome measured are freedom from AF and safety (every complication that occur with ablation) and procedure time, RF time and fluoroscopy time. For the specific exclusion criteria, including (1) inappropriate and irrelevant title or abstract; (2) irretrievable full-texts; (3) comparing the ablation strategies (ablation lesion box set); (4) review article, case report, case series, or conference abstract; and (5) animal or in vitro study.

**Quality Assessment**

Risk of bias for randomized controlled trials was assessed using the revised Cochrane risk-of-bias tool for randomized trials (RoB 2). This tool evaluates five domains of potential bias: (1) bias arising from the randomisation process; (2) bias due to deviations from intended interventions; (3) bias due to missing outcome data; (4) bias in measurement of the outcome; and (5) bias in selection of the reported result. For each domain, signalling questions guide the judgement, which is expressed as low risk of bias, some concerns, or high risk of bias. An overall risk-of-bias judgement is then derived for each trial: studies were classified as at low risk of bias when all domains were judged at low risk; as having some concerns when at least one domain raised some concerns but none was at high risk; and as at high risk of bias when at least one domain was judged at high risk or when multiple domains with some concerns were considered sufficient to lower confidence in the results.

For non-randomized comparative studies, risk of bias was evaluated using the Risk Of Bias In Non-randomized Studies–of Interventions (ROBINS-I) tool. ROBINS-I considers seven domains: (1) bias due to confounding; (2) bias in selection of participants into the study; (3) bias in classification of interventions; (4) bias due to deviations from intended interventions; (5) bias due to missing data; (6) bias in measurement of outcomes; and (7) bias in selection of the reported result. As with RoB 2, domain-level judgements are based on structured signalling questions. Each domain is initially rated as low, moderate, serious, critical risk of bias, or no information; for the purposes of graphical presentation and synthesis, these categories were subsequently collapsed into low risk, some concerns (corresponding to moderate risk), and high risk (including serious and critical risk). An overall ROBINS-I judgement was then assigned to each non-randomized study according to the most severe level of bias in any domain: studies were considered at low risk of bias if all domains were rated low risk; at moderate risk (summarised as some concerns) if at least one domain was at moderate risk but none at serious or critical risk; and at high risk if any domain was rated serious or critical or if multiple domains with moderate risk were expected to materially affect confidence in the estimated treatment effects.

**Atrial arrhythmia recurrence**

For each study, we extracted the definition of atrial arrhythmia recurrence used for the primary efficacy endpoint. Whenever possible, recurrence was defined as any documented episode of atrial fibrillation, atrial tachycardia or atrial flutter lasting ≥30 seconds and occurring after completion of the blanking period prespecified by the investigators. When several arrhythmic endpoints were reported, we systematically prioritised the most comprehensive definition (AF/AT/AFL) over AF alone and the endpoint aligned with the main efficacy analysis of the original trial or cohort.

We also recorded whether a blanking period was applied, its duration, and the type and intensity of rhythm monitoring (scheduled ECGs, Holter monitoring, external or implantable event recorders). In studies where early and late recurrences were not clearly distinguished, recurrence was coded according to the primary outcome definition chosen by the original investigators, and this heterogeneity was acknowledged as a limitation in the interpretation of pooled results. When data were reported as “freedom from arrhythmia” at a given time point, we converted these proportions into the corresponding number of patients with recurrence for the purposes of the rate-based network meta-analysis.

**Procedural complications**

For safety, we extracted all acute periprocedural complications explicitly attributed to the AF ablation procedure and reported at the patient level. In line with the original studies, complications typically included pericardial effusion/tamponade, stroke or transient ischaemic attack, vascular access complications, phrenic nerve injury, atrio-oesophageal fistula, and other events requiring medical or interventional treatment or prolongation of hospital stay. When authors reported both major and minor events (for example, access-site haematomas not requiring intervention), we recorded all events and used the composite of all reported complications as the primary safety outcome in order to maximise statistical power, noting that the exact classification of minor vascular events varied across studies.

Because the time window used to define “periprocedural” complications differed slightly between trials (in-hospital vs. up to 30 days after the procedure), we accepted each study’s original definition and did not attempt to recode events to a uniform time frame. This heterogeneity in outcome definitions was considered a potential source of residual variability and is reflected in the width of the credible intervals for safety estimates.

**Stastistical considerations**

Rankings should be interpreted in conjunction with effect estimates and their uncertainty; they are not intended to support clinical recommendations when credible intervals overlap substantially.

**Table S1. Search Strategies**

| Research motor | code |
| --- | --- |
| PubMed | ( "Atrial Fibrillation"[Mesh] OR "atrial fibrillation"[tiab] OR AF[tiab])  AND  ( "Catheter Ablation"[Mesh] OR "catheter ablation"[tiab] OR "radiofrequency ablation"[tiab] OR "radiofrequency catheter ablation"[tiab])  AND  ( "high-power"[tiab] OR "high power"[tiab]  OR "very high-power"[tiab] OR "very high power"[tiab]  OR "short-duration"[tiab] OR "short duration"[tiab]  OR "low-power"[tiab] OR "low power"[tiab]  OR "long-duration"[tiab] OR "long duration"[tiab]) |
| Cochrane Library | ( [mh "Atrial Fibrillation"] OR "atrial fibrillation":ti,ab,kw OR AF:ti,ab,kw)  AND  ( [mh "Catheter Ablation"] OR "catheter ablation":ti,ab,kw OR "radiofrequency ablation":ti,ab,kw)  AND  ( "high-power":ti,ab,kw OR "high power":ti,ab,kw  OR "very high-power":ti,ab,kw OR "very high power":ti,ab,kw  OR "short-duration":ti,ab,kw OR "short duration":ti,ab,kw  OR "low-power":ti,ab,kw OR "low power":ti,ab,kw  OR "long-duration":ti,ab,kw OR "long duration":ti,ab,kw) |
| Embase | ( 'atrial fibrillation'/exp OR 'atrial fibrillation':ti,ab,kw OR AF:ti,ab,kw)  AND  ( 'catheter ablation'/exp OR 'radiofrequency ablation'/exp  OR "catheter ablation":ti,ab,kw OR "radiofrequency ablation":ti,ab,kw)  AND  ( "high-power":ti,ab,kw OR "high power":ti,ab,kw  OR "very high-power":ti,ab,kw OR "very high power":ti,ab,kw  OR "short-duration":ti,ab,kw OR "short duration":ti,ab,kw  OR "low-power":ti,ab,kw OR "low power":ti,ab,kw  OR "long-duration":ti,ab,kw OR "long duration":ti,ab,kw) |
| Web of Science | TS=("atrial fibrillation")  AND TS=("catheter ablation" OR "radiofrequency ablation")  AND TS=(  "high-power" OR "high power"  OR "very high-power" OR "very high power"  OR "short-duration" OR "short duration"  OR "low-power" OR "low power"  OR "long-duration" OR "long duration") |
| Scopus | TITLE-ABS-KEY("atrial fibrillation")  AND TITLE-ABS-KEY("catheter ablation" OR "radiofrequency ablation")  AND TITLE-ABS-KEY( "high-power" OR "high power"  OR "very high-power" OR "very high power"  OR "short-duration" OR "short duration"  OR "low-power" OR "low power"  OR "long-duration" OR "long duration") |

**Table S2. PRISMA NMA Checklist of Items to Include When Reporting A Systematic Review Involving A Network Meta-Analysis**

| **Section/Topic** | **Item #** | **Checklist Item** | **Reported on Page #** |
| --- | --- | --- | --- |
| **TITLE** |  |  |  |
| Title | 1 | Identify the report as a systematic review *incorporating a network meta-analysis (or related form of meta-analysis).* | ***1*** |
|  |  |  |  |
| **ABSTRACT** |  |  |  |
| Structured summary | 2 | Provide a structured summary including, as applicable:  **Background:** main objectives  **Methods:** data sources; study eligibility criteria, participants, and interventions; study appraisal; and *synthesis methods, such as network meta-analysis.*  **Results:** number of studies and participants identified; summary estimates with corresponding confidence/credible intervals; *treatment rankings may also be discussed. Authors may choose to summarize pairwise comparisons against a chosen treatment included in their analyses for brevity.*  **Discussion/Conclusions:** limitations; conclusions and implications of findings.  **Other:** primary source of funding; systematic review registration number with registry name. | 3 |
|  |  |  |  |
| **INTRODUCTION** |  |  |  |
| Rationale | 3 | Describe the rationale for the review in the context of what is already known*, including mention of why a network meta-analysis has been conducted.* | ***4*** |
| Objectives | 4 | Provide an explicit statement of questions being addressed, with reference to participants, interventions, comparisons, outcomes, and study design (PICOS). | 4 |
|  |  |  |  |
| **METHODS** |  |  |  |
| Protocol and registration | 5 | Indicate whether a review protocol exists and if and where it can be accessed (e.g., Web address); and, if available, provide registration information, including registration number. | 4 |
| Eligibility criteria | 6 | Specify study characteristics (e.g., PICOS, length of follow-up) and report characteristics (e.g., years considered, language, publication status) used as criteria for eligibility, giving rationale. *Clearly describe eligible treatments included in the treatment network, and note whether any have been clustered or merged into the same node (with justification).* | ***5*** |
| Information sources | 7 | Describe all information sources (e.g., databases with dates of coverage, contact with study authors to identify additional studies) in the search and date last searched. | 4 |
| Search | 8 | Present full electronic search strategy for at least one database, including any limits used, such that it could be repeated. | 4 |
| Study selection | 9 | State the process for selecting studies (i.e., screening, eligibility, included in systematic review, and, if applicable, included in the meta-analysis). | 5 |
| Data collection process | 10 | Describe method of data extraction from reports (e.g., piloted forms, independently, in duplicate) and any processes for obtaining and confirming data from investigators. | 5-6 |
| Data items | 11 | List and define all variables for which data were sought (e.g., PICOS, funding sources) and any assumptions and simplifications made. | 5-6, Table S3 |
| **Geometry of the network** | **S1** | Describe methods used to explore the geometry of the treatment network under study and potential biases related to it. This should include how the evidence base has been graphically summarized for presentation, and what characteristics were compiled and used to describe the evidence base to readers. | ***5-6*** |
| Risk of bias within individual studies | 12 | Describe methods used for assessing risk of bias of individual studies (including specification of whether this was done at the study or outcome level), and how this information is to be used in any data synthesis. | 6 |
| Summary measures | 13 | State the principal summary measures (e.g., risk ratio, difference in means). *Also describe the use of additional summary measures assessed, such as treatment rankings and surface under the cumulative ranking curve (SUCRA) values, as well as modified approaches used to present summary findings from meta-analyses.* | 6-7, supplementary |
| Planned methods of analysis | 14 | Describe the methods of handling data and combining results of studies for each network meta-analysis. This should include, but not be limited to:   - *Handling of multi-arm trials;* - *Selection of variance structure;* - *Selection of prior distributions in Bayesian analyses; and* - *Assessment of model fit.* | 6-7 |
| **Assessment of Inconsistency** | **S2** | Describe the statistical methods used to evaluate the agreement of direct and indirect evidence in the treatment network(s) studied. Describe efforts taken to address its presence when found. | 6-7 |
| Risk of bias across studies | 15 | Specify any assessment of risk of bias that may affect the cumulative evidence (e.g., publication bias, selective reporting within studies). | **6-7** |
| Additional analyses | 16 | Describe methods of additional analyses if done, indicating which were pre-specified. This may include, but not be limited to, the following:   - Sensitivity or subgroup analyses; - Meta-regression analyses; - *Alternative formulations of the treatment network; and* - *Use of alternative prior distributions for Bayesian analyses (if applicable).* | ***6-7*** |
|  |  |  |  |
| **RESULTS†** |  |  |  |
| Study selection | 17 | Give numbers of studies screened, assessed for eligibility, and included in the review, with reasons for exclusions at each stage, ideally with a flow diagram. | 8, Figure 1 |
| **Presentation of network structure** | **S3** | Provide a network graph of the included studies to enable visualization of the geometry of the treatment network. | ***8*** |
| **Summary of network geometry** | **S4** | Provide a brief overview of characteristics of the treatment network. This may include commentary on the abundance of trials and randomized patients for the different interventions and pairwise comparisons in the network, gaps of evidence in the treatment network, and potential biases reflected by the network structure. | ***9, Figure2*** |
| Study characteristics | 18 | For each study, present characteristics for which data were extracted (e.g., study size, PICOS, follow-up period) and provide the citations. | 8-9, Figure S1-S2 |
| Risk of bias within studies | 19 | Present data on risk of bias of each study and, if available, any outcome level assessment. | 8-9, Figure S1-S2 |
| Results of individual studies | 20 | For all outcomes considered (benefits or harms), present, for each study: 1) simple summary data for each intervention group, and 2) effect estimates and confidence intervals. *Modified approaches may be needed to deal with information from larger networks.* | 9, Table 1 |
| Synthesis of results | 21 | Present results of each meta-analysis done, including confidence/credible intervals. *In larger networks, authors may focus on comparisons versus a particular comparator (e.g. placebo or standard care), with full findings presented in an appendix. League tables and forest plots may be considered to summarize pairwise comparisons.* If additional summary measures were explored (such as treatment rankings), these should also be presented. | ***9-10, Table 2-3*** |
| **Exploration for inconsistency** | **S5** | Describe results from investigations of inconsistency. This may include such information as measures of model fit to compare consistency and inconsistency models, *P* values from statistical tests, or summary of inconsistency estimates from different parts of the treatment network. | ***10, Figure 5, Table 4-5, S4-S5-S6-S7*** |
| Risk of bias across studies | 22 | Present results of any assessment of risk of bias across studies for the evidence base being studied. | 11, Figure S1-S2, Table 5 |
| Results of additional analyses | 23 | Give results of additional analyses, if done (e.g., sensitivity or subgroup analyses, meta-regression analyses*, alternative network geometries studied, alternative choice of prior distributions for Bayesian analyses,* and so forth). | ***10-12, Table 4-5*** |
|  |  |  |  |
| **DISCUSSION** |  |  |  |
| Summary of evidence | 24 | Summarize the main findings, including the strength of evidence for each main outcome; consider their relevance to key groups (e.g., healthcare providers, users, and policy-makers). | 10-14 |
| Limitations | 25 | Discuss limitations at study and outcome level (e.g., risk of bias), and at review level (e.g., incomplete retrieval of identified research, reporting bias). *Comment on the validity of the assumptions, such as transitivity and consistency. Comment on any concerns regarding network geometry (e.g., avoidance of certain comparisons).* | 14-15 |
| Conclusions | 26 | Provide a general interpretation of the results in the context of other evidence, and implications for future research. | 15-16 |
|  |  |  |  |
| **FUNDING** |  |  |  |
| Funding | 27 | Describe sources of funding for the systematic review and other support (e.g., supply of data); role of funders for the systematic review. This should also include information regarding whether funding has been received from manufacturers of treatments in the network and/or whether some of the authors are content experts with professional conflicts of interest that could affect use of treatments in the network. | ***N.A*** |

**Supplementary Table S3** PICO

| **Components of PICO** | **Definition** |
| --- | --- |
| Population | Patients with atrial fibrillation |
| Intervention | - High power short duration (HPSD) RF  - Very high power short duration (vHPSD) RF |
| Comparison | Low power long duration (LPLD) RF |
| Outcome | - Primary Outcome (Effecacy): Freedom from atrial arrhythmia  - Secondary Outcome (Safety): Every complica­tion that occur with ablation and during hospitalization  - Secondary Outcome (Efficiency): procedure time, RF delivery time, fluoroscopy time |

PICO, Population, Intervention, Comparison, Outcome; LPLD, low power long duration; HPSD, high power short duration; vHPSD, very high power short duration, RF radiofrequency

**Baseline information**

**Supplementary Table S4** Adjunctive procedural protocol features of the studies included in the network meta-analysis, stratified by ablation strategy (low-power long-duration [LPLD], high-power short-duration [HPSD] and very-high-power short-duration [vHPSD] radiofrequency [RF] ablation).

|  | **Power (W)** | **Application time** | **Intraprocedural waiting time (min)** | **Intraprocedural evaluation of isolation** | **Esophageal monitor/marker** | **Ablation strategy** | **Irrigation** | **Contact force** | **Index of ablation** | **Procedure time (min)** |
| --- | --- | --- | --- | --- | --- | --- | --- | --- | --- | --- |
| Mansour et al. | 25/50 | 30/12-15s | - | Bidirectional block | No | PVI only (segmental vs WACA) | No | No | No | -/- |
| Nilsson et al. | 30/45 | <120/20s | 30 | EGM | No | PVI only | Yes | No | No | -/- |
| Yamada et al. | 30/40 | Signal abolition | - | PVI block | No | PVI only (segmental) | No | No | No | 223/152 |
| Kanj et al. | 35/45 | multiparameter | - | Bidirectional block | Yes | PVI only | Yes | No | No | -/- |
| Matiello et al. | 30/40 | Signal abolition | - | EGM | No | PVI + ML | Mixed | No | No | -/- |
| Winkle et al. | 40/70 | 20-45/10s | - | EGM/entrance block + isoprotenerol test | Yes | PVI + fragmented | Yes | No | No | 129/217 |
| Baher et al. | 25/50 | 10-35/5s | - | Bidirectional block | Yes | PVI (+line in some cases) | Yes | Yes | No | 251/149 |
| Okamatsu et al. | 20/50 | AI/AI | - | Bidirectional block + isoprotenerol | Yes | PVI (+line in some cases) | Yes | Yes | AI | 163/- |
| Pambrun et al. | 25/50 | Signal abolition+5/2s | 20 | Bidirectional block | No | PVI only | Yes | Yes | No | 107/73 |
| Bunch et al. | 30/50 | 10-20/5/15s | - | Bidirectional block | Yes | PVI (+line in some cases) | Yes | Mixed | No | 171/104 |
| Ejima et al. | 30/50 | Signal abolition+5s | 20 | Bidirectional block + adenosine +isoprotenerol tests | No | PVI only | Yes | Yes | AI | 140/119 |
| Kottmaier et al. | 30/70 | 20-40/7s | 20 | EGM + adenosine test | No | PVI only | Yes | No | No | 111/90 |
| Kumagai et al. | 20/50 | 30/5s | - | Isoprotenerol test | Yes | PVI + posterior box | Yes | No | No | 85/65 |
| Kyriakopoulou et al. | 35/40 | AI/AI | - | Adenosine test | Yes | PVI only | Yes | Yes | AI | 111/91 |
| Leo et al. | 20/40 | LSI/LSI | 30 | Bidirectional block | Yes | PVI only | Yes | Yes | LSI | 180/165 |
| Shin et al. | 30/50 | 40/10s | - | Bidirectional block | No | PVI (+line in some cases) | Yes | Yes | AI | 162/122 |
| Yavin et al. | 20/45 | 20-30/8-15s | 20 | Bidirectional block + adenosine +isoprotenerol tests | Yes | PVI (+line in some cases) | Yes | Yes | No | -/- |
| Yazaki et al. | 20/50 | 15-30/5-12s | 20 | Bidirectional block + adenosine +isoprotenerol tests | Yes | PVI only | Yes | Yes | AI | 150/115 |
| Chen et al. | 25/40 | AI/10s | 30 | EGM + adenosine +isoprotenerol tests | No | PVI only | Yes | Yes | AI | 124/91 |
| Dikdan et al. | 20/50 | 30-60/15s +LSI | - | Bidirectional block | Yes | PVI only | Yes | Yes | LSI | 101/71 |
| Francke et al. | 20/50 | AI/AI | - | Bidirectional block | No | PVI (+line in some cases) | Yes | Yes | AI | 109/80 |
| Hansom et al. | 20/50 | 20-40/6/10s | 20-30 | Bidirectional block + adenosine test | Some cases | PVI (+line in some cases) | Yes | Yes | AI | 309/229 |
| O'Brien et al. | 35/50 | AI/AI | - | Bidirectional block | Yes | PVI (+line in some cases) | Yes | Yes | AI | 140/121 |
| Okamatsu et al. | 20/40 | AI/AI | - | Bidirectional block + isoprotenerol test | No | PVI (+line in some cases) | Yes | Yes | AI | 180/153 |
| Park et al. | 20/40 | AI/10-15s | 30 | Bidirectional block + isoprotenerol test | Yes | PVI + additional line in persistent AF | Yes | Yes | AI | 181/135 |
| Wielandts et al. | 35/45 | AI/AI | - | Bidirectional block + adenosine test | Yes | PVI (+ICT if typical Fla documented) | Yes | Yes | AI | 103/85 |
| Cheng et al. | 30/45 | 20-40/10/20s | - | Bidirectional block | No | PVI only mainly | Yes | Yes | AI | 145/112 |
| Cui et al. | 30/50 | AI/AI | 30 | Bidirectional block + adenosine +isoprotenerol tests | No | PVI (+line in some cases) | Yes | Yes | AI | -/- |
| Ding et al. | 35/50 | AI/AI | - | Bidirectional block | Yes | PVI + posterior box | Yes | Yes | AI | 143/120 |
| Hijioka et al. | 20/45 | AI/AI | - | Bidirectional block + adenosine +isoprotenerol tests | No | PVI only | Yes | Yes | AI | 193/172 |
| Liu et al. | 35/45 | AI/AI | 30 | Entrance block + adenosine/isoprotenerol test | No | PVI only | Yes | Yes | AI | 34/25 |
| Mueller et al. | 50/90 | AI/4s | 30 | Entrance block | No | PVI only | Yes | Yes | AI | 85/89 |
| Sallo et al. | 30/50/90 | AI/AI/4s | 30 | Bidirectional block + adenosine test | No | PVI only | Yes | Yes | AI | 85/79/70 |
| Seidl et al. | 50/90 | AI/4s | - | Bidirectional block | No | PVI only | Yes | Yes | AI | 121/106 |
| Vassallo et al. | 20/50 | LSI/4s | - | Bidirectional block + adenosine test | Yes | PVI only | Yes | Yes | LSI | 145/92 |
| Heeger et al. | 40/90 | AI/4s | - | Isolation of single PV | Yes | PVI (+ICT in some cases) | Yes | Yes | AI | 101/59 |
| Jin et al. | 25/40 | AI/AI (<30s) | - | First passo isolation only | No | PVI (+line in some cases) | Yes | Yes | AI/LSI | -/- |
| Lee et al. | 25/50 | AI/AI | - | Bidirectional block + adenosine test | Yes | PVI only | Yes | No | AI | 286/236 |
| Manukyan et al. | 20/50 | LSI/11-13s | - | First passo isolation only | Some | PVI only | Yes | Yes | LSI | 98/73 |
| O'Neill et al. | 40/90 | AI/4s | - | Bidirectional block + adenosine test | Yes | PVI only | Yes | Yes | AI | 75/70 |
| Popa et al. | 30/70 | 15-30/5/10s | 20 | Bidirectional block + adenosine test | No | PVI (+line in some cases) | Yes | Yes | AI | 155/122 |
| Sousa et al. | 25/40 | AI/AI | - | Bidirectional block + adenosine test | No | PVI only | Yes | Yes | AI | 100/80 |
| Zhu et al. | 30/40 | AI/AI | - | - | No | PVI only | Yes | Yes | LSI | -/- |
| Bortone et al. | 50/90 | AI/4s | 20 | Bidirectional block + adenosine test | Yes | PVI only | Yes | Yes | AI | 65/61 |
| Compagnucci et al. | 35/90 | AI/4s | 20 | Bidirectional block | Yes | PVI + PW ablation | Yes | Yes | AI | 198/81 |
| Fink et al. | 30/50/90 | AI/AI/4s | - | Bidirectional block | No | PVI (+ICT if typical Fla documented) | Yes | Yes | AI | 105/81/70 |
| Joza et al. | 25/40 | AI/AI | 20 | Bidirectional block + adenosine test | No | PVI only | Yes | Yes | AI/LSI | 254/152 |
| Keegan et al. | 30/70 | LSI/9s | - | - | No | PVI only | Yes | Yes | AI/LSI | 180/125 |
| Mitrzak et al. | 35/90 | AI/4s | 20 | Entrance block | No | PVI only | Yes | Yes | AI | 145/120 |
| Szegedi et al. | 50/90 | AI/4s | 20 | Bidirectional block | No | PVI only | Yes | Yes | AI | 84/76 |
| Yazaki et al. | 30/50 | 5-10/3-6s | 20 | Bidirectional block + adenosine +isoprotenerol tests | No | PVI only | Yes | Yes | No | 169/123 |

**Sensitivity analyses section**

**Supplementary Table S5** Global fit (Consistent vs UME)

| **Model** | **Residual deviance** | **pD** | **DIC** |
| --- | --- | --- | --- |
| Consistency | 115.4 (104 pts) | 69.6 | 184.9 |
| UME | 115.0 (104 pts) | 70.5 | 185.5 |

**Supplementary Table S6** Local inconsistency (node-split p for omega)

| **Comparison** | **P value** |
| --- | --- |
| HPSD vs LPLD | 0.274 |
| vHPSD vs LPLD | 0.262 |
| vHPSD vs HPSD | 0.610 |

**Supplementary Table S7** Egger test

| **Term** | **Estimate** | **SE** | **p-value** |
| --- | --- | --- | --- |
| Intercept | 0.1535 | 0.0620 | 0.0165 |
| seTE slope | -0.7960 | 0.2614 | 0.0036 |

**Supplementary Table S8** Leave-one-out analysis

| **Contrast** | **RR min** | **RR max** | **N of fits** | **RR base** | **2.5%** | **97.5%** |
| --- | --- | --- | --- | --- | --- | --- |
| HPSD vs LPLD | 0.835 | 0.872 | 51 | 0.851 | 0.739 | 0.958 |
| vHPSD vs LPLD | 0.759 | 0.831 | 51 | 0.785 | 0.630 | 0.948 |

**Supplementary Figures**

**Supplementary Figure S1**. Risk of bias assessment of randomized controlled trials according to the revised Cochrane risk-of-bias tool for randomized trials (RoB 2). The left panel displays domain-level judgements for each included trial across the five RoB 2 domains (randomisation process, deviations from intended interventions, missing outcome data, measurement of the outcome, and selection of the reported result), with green, yellow, and red symbols indicating low risk of bias, some concerns, and high risk of bias, respectively. The right panel summarizes the distribution of these judgements as stacked horizontal bar charts showing the percentage of trials rated at low risk, some concerns, or high risk of bias in each domain.


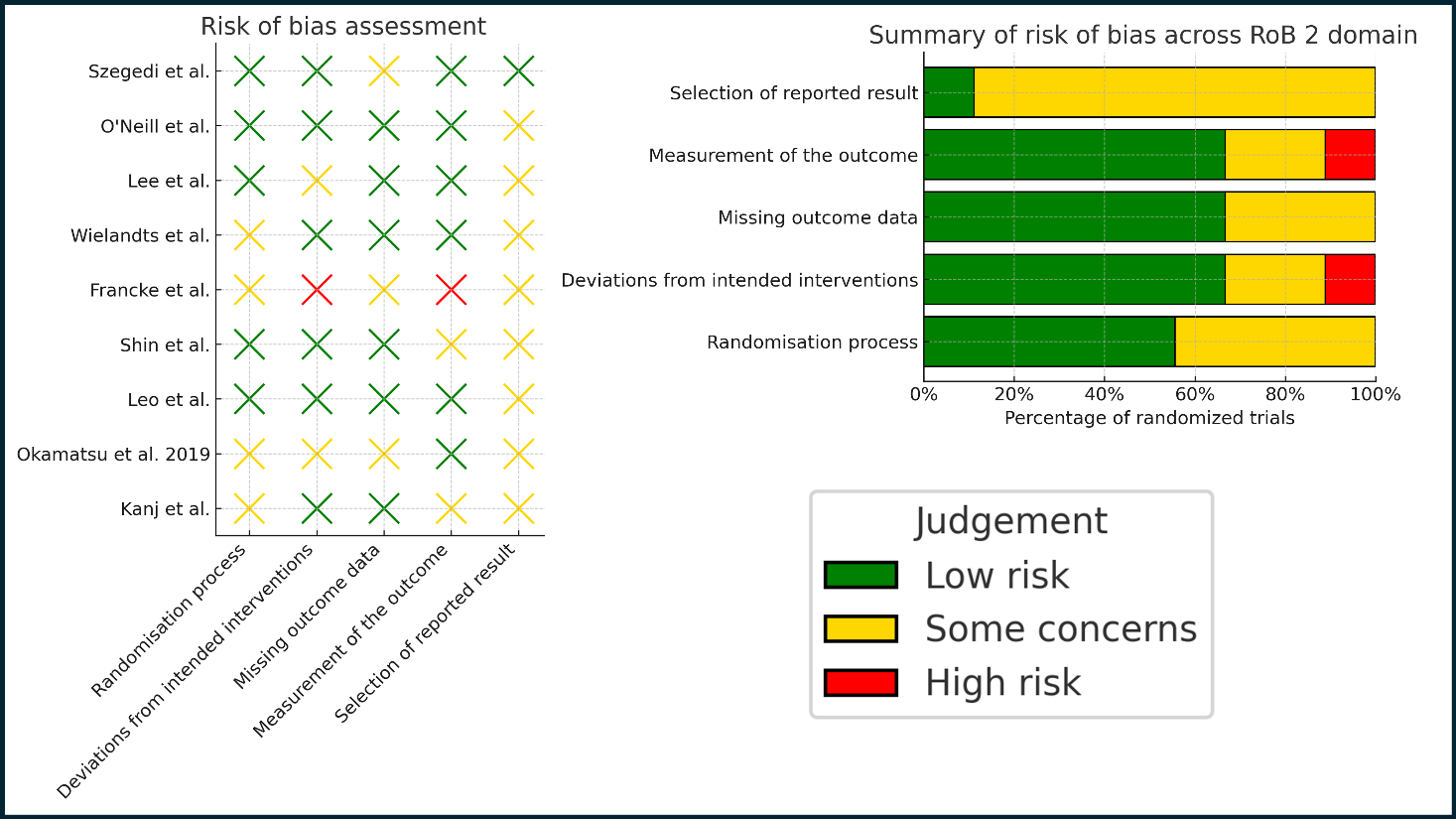


**Supplementary Figure S2**. Risk of bias assessment of non-randomized studies according to the Risk Of Bias In Non-randomized Studies–of Interventions (ROBINS-I) tool. The left panel displays domain-level judgements for each included study across the seven ROBINS-I domains (bias due to confounding, bias in selection of participants, bias in classification of interventions, bias due to deviations from intended interventions, bias due to missing data, bias in measurement of outcomes, and bias in selection of reported results), with green, yellow, and red symbols indicating low risk of bias, some concerns, and high risk of bias, respectively. The right panel summarizes the distribution of these judgements as stacked horizontal bar charts showing the percentage of non-randomized studies rated at low risk, some concerns, or high risk of bias in each domain.


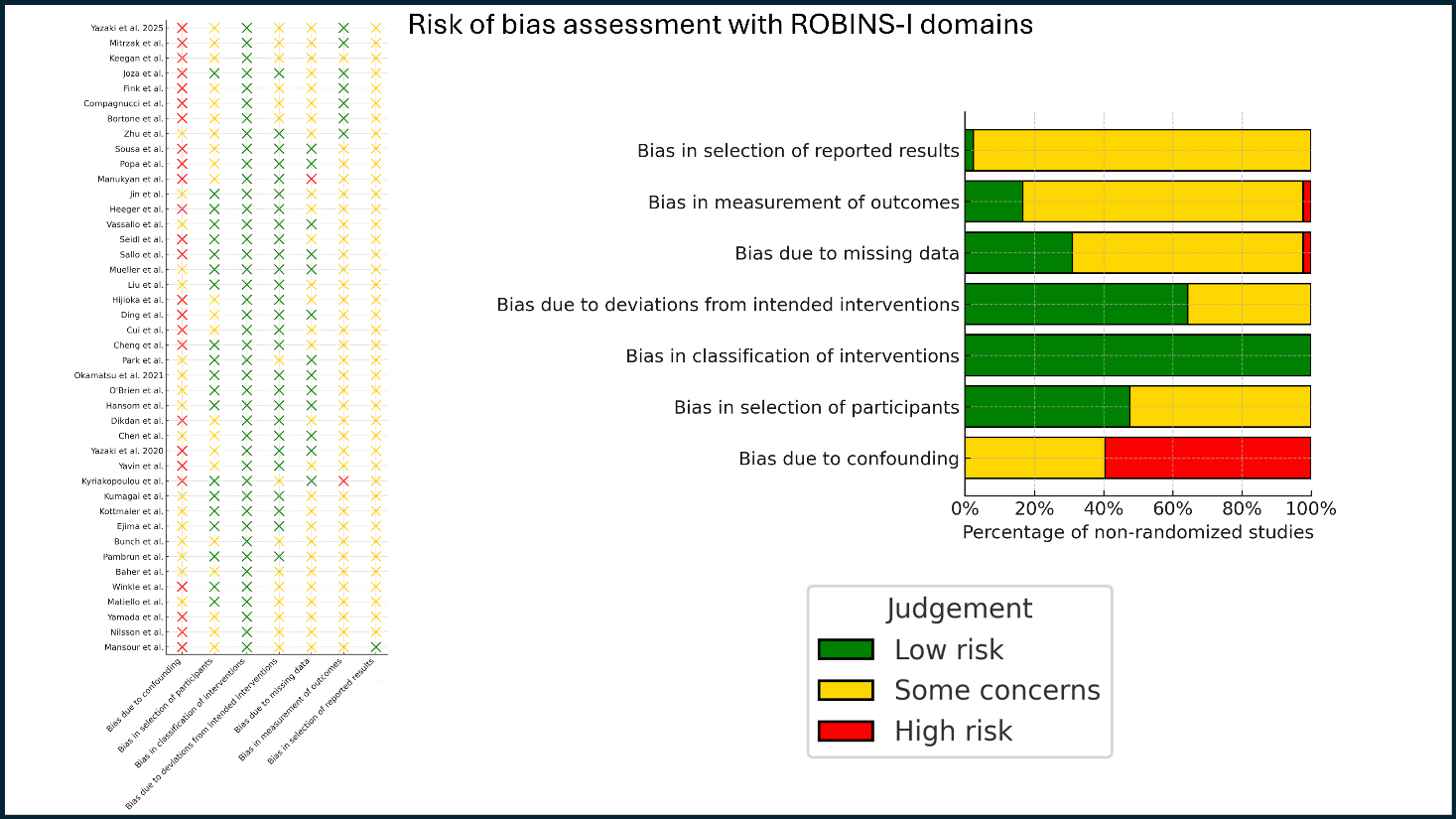

Supplement: Supplementary Material [file mmc1.docx]
